# First person – Armando Cortés-Reséndiz

**DOI:** 10.1242/dmm.053137

**Published:** 2026-09-17

## Abstract

First Person is a series of interviews with the first authors of a selection of papers published in Disease Models & Mechanisms, helping researchers promote themselves alongside their papers. Armando Cortés-Reséndiz is first author on ‘A bioengineered platform reveals progressive impairment of skeletal muscles by metabolic dysfunction-associated steatotic liver disease-derived factors’, published in DMM. Armando is a PhD student in the lab of Javier Ramón-Azcón at Institute for Bioengineering of Catalonia (IBEC), Barcelona, Spain, developing human *in vitro* models to investigate complex disease mechanisms and organ–organ interactions, with the goal of making biomedical research more predictive and translational.

**Figure:**
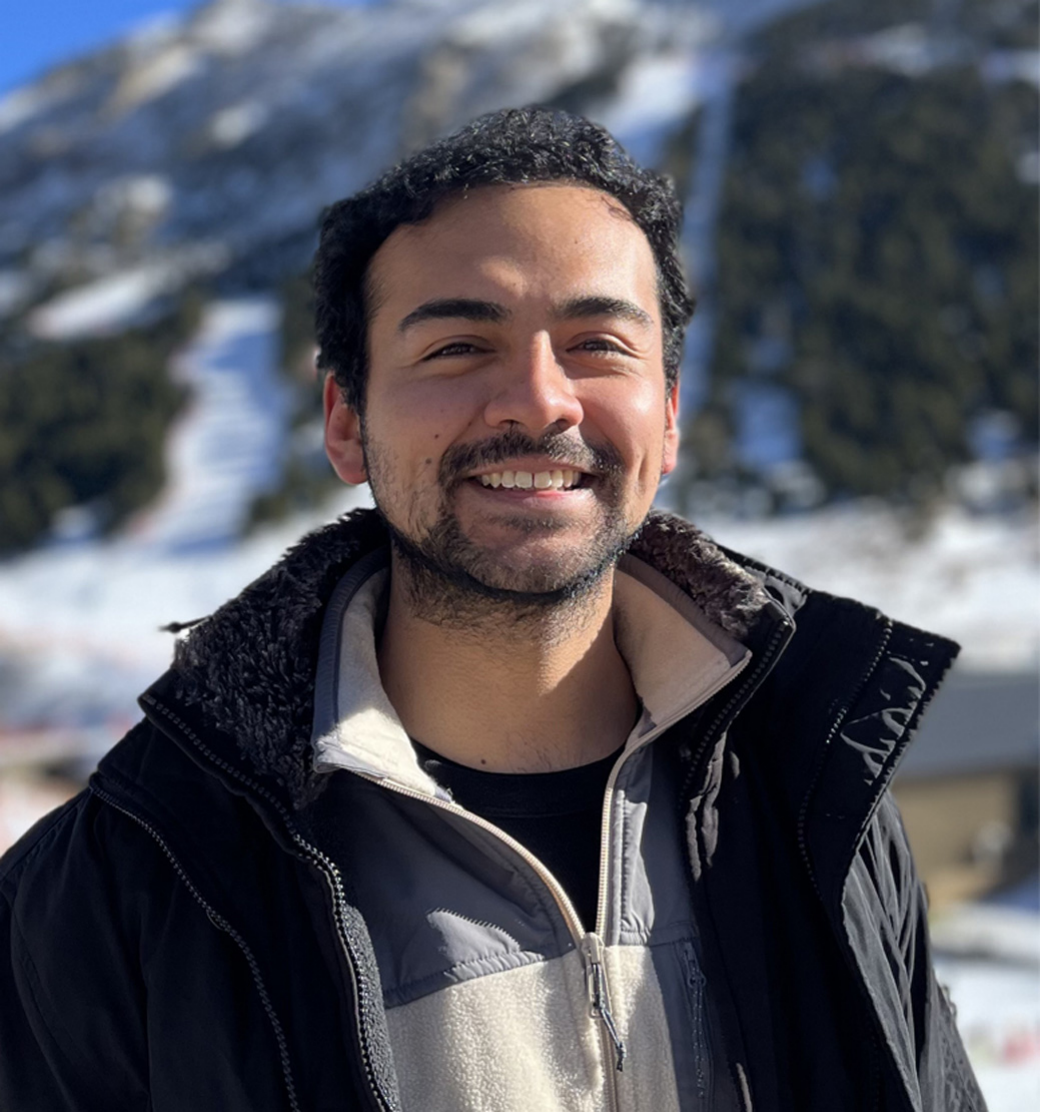
Armando Cortés-Reséndiz

**Who or what inspired you to become a scientist?**

My mother is a chemist, and my uncle is an engineer; both contributed to my early fascination with science. As a kid, I remember my mother showing me how pH indicators change colour during titration reactions and being amazed that science could be so visual. My uncle, meanwhile, shared his passion for biology and zoology with me, buying me books and animal documentaries throughout my childhood. Whenever I struggled with mathematics, I had an expert at home who seemed to know the answer to everything. Growing up around their curiosity made science feel more like a way of understanding the world.

**What is the main question or challenge in disease biology you are addressing in this paper? How did you go about investigating your question or challenge?**

We wanted to understand whether factors released by a diseased liver can directly impair skeletal muscle, independently of the many other variables that coexist in patients. To investigate this, we first engineered human 3D liver tissues that reproduced features of different stages of metabolic dysfunction-associated steatotic liver disease (MASLD), from steatosis to a more severe metabolic dysfunction-associated steatohepatitis-like state. We then transferred the environment generated by these liver tissues to bioengineered skeletal muscles and measured how their structure, molecular regulation and, importantly, their ability to contract were affected. From there, I had to follow a more exploratory strategy, since I was not sure how the skeletal muscle would react. The unexpected behaviour of the muscle made the exploratory part of the project especially interesting and rewarding.

**How would you explain the main findings of your paper to non-scientific family and friends?**

In MASLD, the liver accumulates a lot of fat, and that is very dangerous in the long term. When you imagine a liver disease, you might be inclined to think the problem stays in the liver. But recent science indicates this is not true. In fact, scientists are realizing that the liver is a very interconnected organ and can have adverse effects on other tissues if it is damaged or is under stress conditions. Clinicians worldwide have noticed that many people with excess fat in the liver lose muscle mass and strength. This is common in people with other diseases like diabetes and can happen for many reasons, for example, a poor diet, a sedentary lifestyle or even just genetic predisposition. It is important that we know how to intervene so we can develop therapies and medicines.

The limitation of science right now is that we cannot study how this relationship works in live humans, and we need to think about how to approximate as much as possible. For this reason, we developed a miniaturized tool using human cells and materials that resemble the ones we have in our own body; this way, we can test what happens if we turn some knobs without experimenting in real humans.

We simulated a diseased liver in our miniaturized tissues and made sure they behaved as a real one, by comparing how much they resemble the typical characteristics of MASLD. Then, we fabricated miniaturized muscles and evaluated how they reacted when being in contact with miniaturized livers. We found that the muscles that had been in contact with diseased livers were a lot weaker than the ones in contact with healthy livers. This means that healthy and diseased livers each send different signals to the muscles, influencing how strong and capable they are. We demonstrated that we could approximate well what happens in a real body with this tool generated in a laboratory.

Recent science indicates that liver disease does not only affect the liver. Our liver constantly communicates with the rest of the body, and, when it becomes unhealthy, the signals it sends can also affect other organs. Doctors have observed that many people with fatty liver disease also lose muscle strength and, in some cases, muscle mass, but it is difficult to determine exactly why and how this damage is caused by the liver.

To study this, we created miniature liver and muscle tissues in the laboratory using human cells. We made the liver tissues progressively more diseased and then exposed the miniature muscles to the substances released by those livers. We found that signals from diseased livers made the muscles weaker, and the more severe the liver disease became, the greater the loss of muscle function. We also realized that muscle quality and performance can deteriorate before major changes in size become apparent. Our model gives us a controlled way to study this communication between organs without experimenting directly in humans.

… we developed a miniaturized tool using human cells and materials that resemble the ones we have in our own body; this way, we can test what happens if we turn some knobs without experimenting in real humans

**What are the potential implications of these results for disease biology and the possible impact on patients?**

Our results provide a controlled way to investigate how signals released by the liver contribute to muscle dysfunction as liver disease progresses. Because the platform separates liver-derived effects from many of the confounding factors present in patients, it could help identify the specific molecules and pathways responsible for liver-related muscle deterioration and reveal new therapeutic targets. It also provides a system for testing whether treatments directed at the liver can prevent or rescue damage to skeletal muscle. In the longer term, this type of human *in vitro* model could contribute to more mechanistically informed therapies and to a better understanding of why patients experience functional muscle decline.

**Figure:**
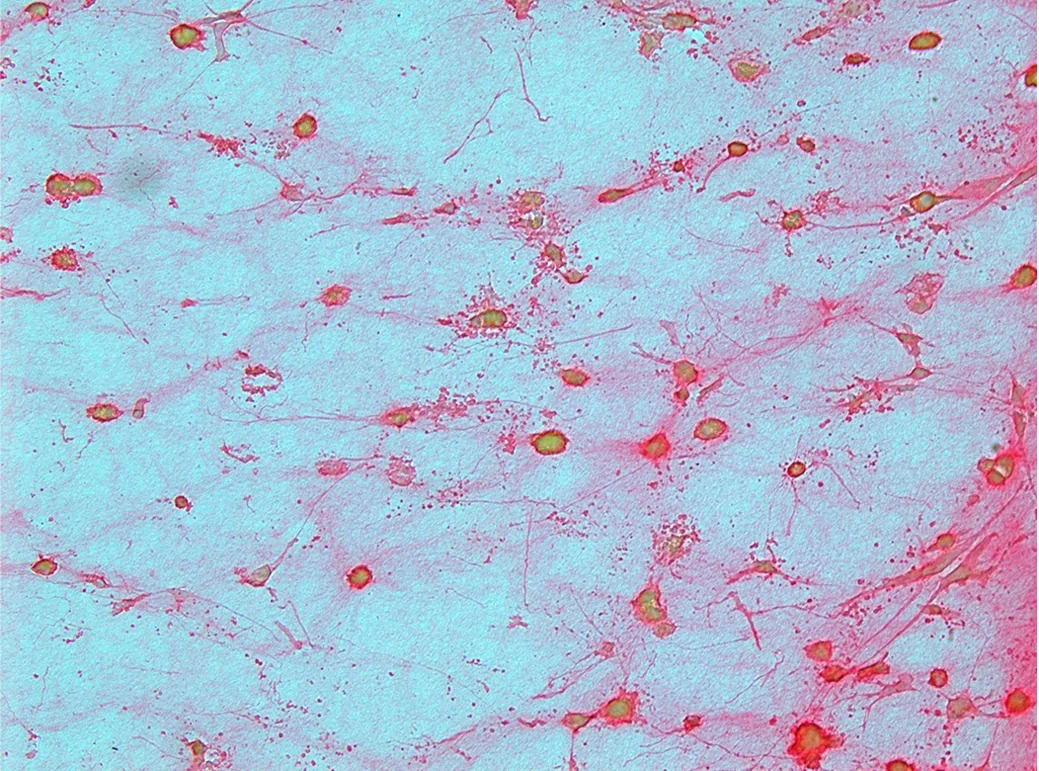
**Representative brightfield image of a Picrosirius Red-stained bioengineered liver construct.** The staining enables visualization of collagen deposition in fibrosis together with the size and morphology of cells embedded within the hydrogel. Cells display heterogeneous sizes and shapes, including some with disrupted or ruptured appearance; cell cytoplasm appears in yellow-golden tones, while deposited collagen fibres are visible in red.

**Why did you choose DMM for your paper?**

DMM was a natural fit because our work sits at the intersection of disease biology, human *in vitro* modelling and functional tissue engineering. This study describes a platform used to investigate the biological links between liver disease and skeletal muscle wasting. I also like DMM's emphasis on experimentally tractable disease models and on connecting findings with their broader relevance to real human disease.

**Given your current role, what challenges do you face and what changes could improve the professional lives of other scientists in this role?**

One of my greatest personal challenges has been building a scientific career far from my home country and family. Opportunities in science are not equally distributed worldwide, and many early-career researchers must move abroad to access good career opportunities. International mobility is extremely enriching, but it also comes with personal, financial and cultural costs that are not always sufficiently acknowledged. I think better long-term career stability and stronger support for international researchers would make a significant difference. I also believe that scientific institutions should continue working towards inclusive environments in which people from different cultural and socioeconomic backgrounds can not only enter science but genuinely feel able to build a sustainable career within it.

**What's next for you?**

I want to continue working at the interface between human *in vitro* models, disease biology and translation. Scientifically, I am particularly interested in investigating whether therapies targeting the diseased liver can prevent or rescue the muscle dysfunction we observed. More broadly, I want to contribute to making advanced *in vitro* platforms more robust, accessible and compatible with real-world applications in biomedical research and drug development. My next career steps will therefore focus on bringing together scientific development and translational impact.

**Tell us something interesting about yourself that wouldn't be on your CV**

One of my biggest passions outside science is dog training. I have recently started exploring competitive obedience and protection sports, which keeps me active and gives me a completely different challenge when I am away from the laboratory. Just like science, good dog training requires careful observation, consistency, adapting your approach when something does not work and breaking a complex goal into small, achievable steps. The difference here is that your training partner has a personality, preferences and ideas of his own, which makes the process much more unpredictable and makes you stay creative.

I do not think [*in vitro* disease models] are by any means ready to replace other types of models, but are valuable as complementary tools, making disease research more mechanistic, human relevant and predictive

**Why are *in vitro* disease models important to tackle the diseases of the present and the future?**

Many human diseases arise from interactions between multiple cell types, organs, environmental factors and individual genetic backgrounds, making them extremely difficult to study with any single experimental approach. Human *in vitro* models allow us to isolate the mechanisms, control experimental variables and observe processes that would be inaccessible in real patients. As these models become more sophisticated, they can also reproduce aspects of human biology that conventional cell cultures cannot capture, while helping to reduce reliance on animal experiments. To this day, I do not think they are by any means ready to replace other types of models, but are valuable as complementary tools, making disease research more mechanistic, human relevant and predictive. Their potential lies in helping us explain why diseases develop, and test which interventions are most likely to work well.
